# Prediction of a native ferroelectric metal

**DOI:** 10.1038/ncomms11211

**Published:** 2016-04-04

**Authors:** Alessio Filippetti, Vincenzo Fiorentini, Francesco Ricci, Pietro Delugas, Jorge Íñiguez

**Affiliations:** 1 CNR-IOM SLACS Cagliari, Istituto Officina dei Materiali, Cittadella Universitaria, Monserrato (CA) 09042-I, Italy; 2 Dipartimento di Fisica, Università di Cagliari, Cittadella Universitaria, Monserrato (CA) 09042-I, Italy; 3 Institute of Condensed Matter and Nanosciences, Université Catholique de Louvain, 1348 Louvain-la-Neuve, Belgium; 4 Istituto Italiano di Tecnologia, Via Morego 30, 16163 Genova, Italy; 5 Scuola Internazionale Superiore di Studi Avanzati, Via Bonomea 265, 34136 Trieste, Italy; 6 Department of Materials Research and Technology, Luxembourg Institute of Science and Technology, 5 Avenue des Hauts-Fourneaux, L-4362 Esch/Alzette, Luxembourg; 7 Institut de Ciència de Materials de Barcelona (ICMAB-CSIC), Campus UAB, 08193 Bellaterra, Spain

## Abstract

Over 50 years ago, Anderson and Blount discussed symmetry-allowed polar distortions in metals, spawning the idea that a material might be simultaneously metallic and ferroelectric. While many studies have ever since considered such or similar situations, actual ferroelectricity—that is, the existence of a switchable intrinsic electric polarization—has not yet been attained in a metal, and is in fact generally deemed incompatible with the screening by mobile conduction charges. Here we refute this common wisdom and show, by means of first-principles simulations, that native metallicity and ferroelectricity coexist in the layered perovskite Bi_5_Ti_5_O_17_. We show that, despite being a metal, Bi_5_Ti_5_O_17_ can sustain a sizable potential drop along the polar direction, as needed to reverse its polarization by an external bias. We also reveal striking behaviours, as the self-screening mechanism at work in thin Bi_5_Ti_5_O_17_ layers, emerging from the interplay between polar distortions and carriers in this compound.

## Abstract (web summary)

Ferroelectricity, spontaneous switchable polarization, is usually deemed incompatible with the electronic screening of a metal. Here, the authors use *ab initio* theory to predict that metallicity natively coexists with ferroelectric polarization and finite depolarizing fields in the perovskite Bi_5_Ti_5_O_17_.

## Graphical abstract

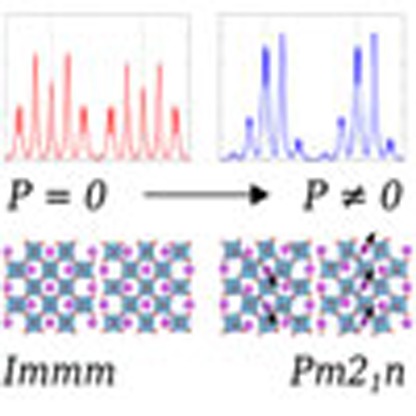

The possibility that metals may exhibit ferroelectricity is an intriguing open issue. Anderson and Blount^1^ showed that certain martensitic transitions involve inversion symmetry breaking and, formally, the existence of a polar axis. Metallic ferroelectric behaviour has thus been claimed for metals undergoing a centrosymmetric (CS) to non-CS structural transformation, such as^2^,^3^ Cd_2_ReO_7_ and LiOsO_3_, or being natively non-CS, such as^4^ (Sr,Ca)Ru_2_O_6_. The same label has been attached to ferroelectric insulators whose polar distortion survives moderate metallicity induced by doping or proximity^5^,^6^,^7^. However, it seems fair to say that none of these systems, nor any other to our knowledge, embodies a truly ferroelectric metal with native switchable polarization and native metallicity coexisting in a single phase.

Here we report a theoretical prediction of such a material. By first-principles calculations, we show that the layered perovskite Bi_5_Ti_5_O_17_ has a non-zero density of states (DOSs) at the Fermi level and metal-like conductivity, as well as a spontaneous polarization in zero field. Further, we predict that the polarization of Bi_5_Ti_5_O_17_ is switchable both in principle (the material complies with the sufficient symmetry requirements) and in practice (in spite of being a metal, Bi_5_Ti_5_O_17_ can sustain a sizable potential drop along the polar direction, as needed to revert its polarization by application of an electric bias). Beyond their conceptual importance, our results reveal striking behaviours—such as a self-screening mechanism at work in thin Bi_5_Ti_5_O_17_ layers—emerging from the intimate interplay between polar distortions and free carriers. Our results thus challenge the common wisdom regarding the possibilities to control charges and fields at the nano-scale, with exciting potential implications in areas ranging from photovoltaics to electronics.

## Results

### Non-CS metallic ground state in Bi_5_Ti_5_O_17_

We focus on layered perovskite titanates A_*n*_Ti_*n*_O_3*n*+2_ (refs 8, 9), whose structure foreshadowing low-dimensional behaviour combines with their tunable conduction charge: assuming fixed ionic charges for A^3+^ and O^2−^, Ti has nominal oxidation state of (3+4/*n*), that is, between 4+ for *n*=4 (for example, the band insulator, high-temperature ferroelectric La_2_Ti_2_O_7_) and 3+ in the *n→∞* limit (for example, the Mott-insulating Ti-3d^1^ perovskite LaTiO_3_). The metallic *n*>4 phases are not nearly as studied as the end compounds^10^,^11^,^12^. Motivated by experimental reports of possible non-CS structures in the *n*=5 compound La_5_Ti_5_O_17_ (La-5517 hereafter)^13^, here we discuss this material as well as the alternative composition Bi_5_Ti_5_O_17_ (Bi-5517).

**Figure 1:**
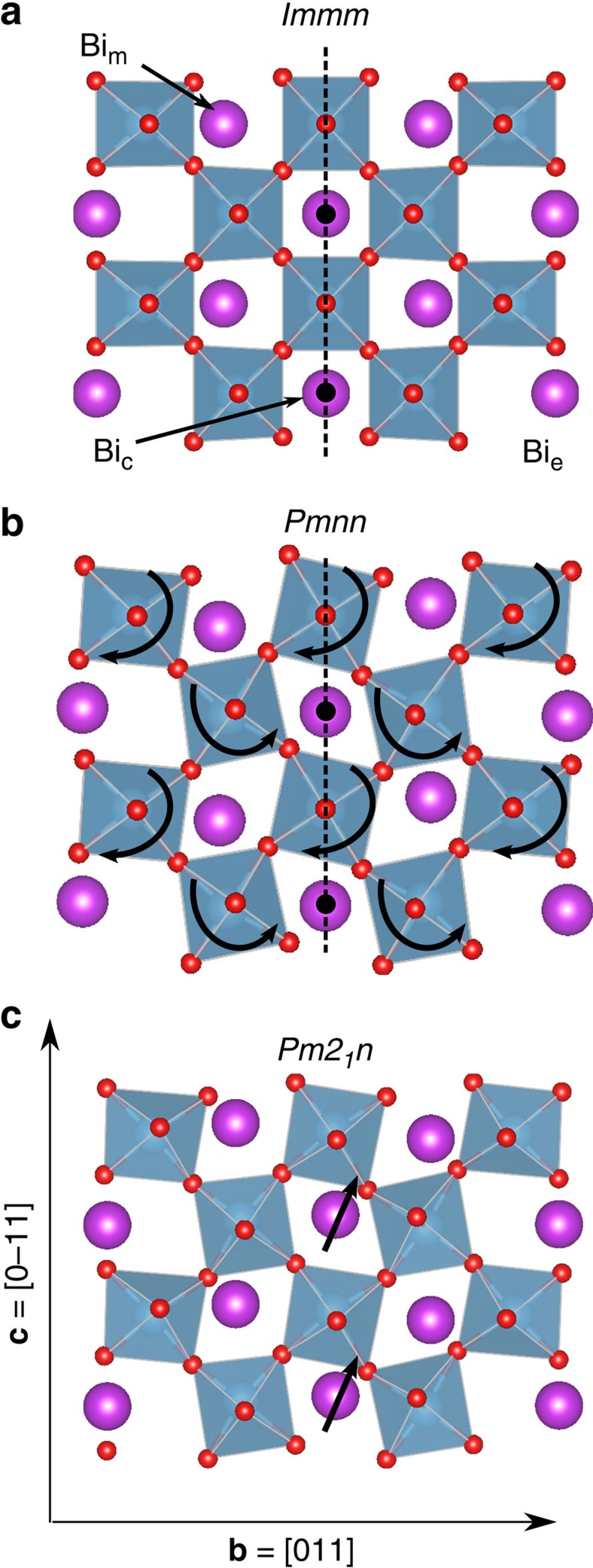
Structural detail of Bi_5_Ti_5_O_17_ phases. Sketch of the Bi-5517 phases discussed in the text, showing relevant structural features. Large violet balls stand for Bi atoms, small red balls for oxygens, green polyhedra for the TiO_6_ groups. Most relevant atomic distortions indicated with arrows. For each structure, black lines outline the supercell profile (**a**) *Immm*, (**b**) *Pmnn*, (**c**) *Pm2*_*1*_*n*. Only one **b**-oriented slab is shown (there are two such slabs in the simulation cell). Bi types indicated in panel (**a**). **b**-oriented mirror plane, and inversion centres at the Bi_c_ positions, indicated in panels (**a**,**b**). In the *Pmnn* case, this mirror plane has an associated ½(**a**+**b**+**c**) glide translation; hence, the symmetry is not obvious from the figure. Bi_c_ off-centering displacements indicated in panel (**c**). The Bi_c_ displacement along **c** is compensated by with a symmetric one occurring in the second slab in the cell (not shown); the Bi_c_ displacement along **b** adds up with the symmetric one in the second slab, and thus a **b**-oriented polarization appears.

The *n*=5 layered titanate can be viewed as a stack of slabs containing 5 [011]-oriented perovskite-like planes and AO terminated. (See Fig. 1, as well as Supplementary Fig. 1 and Supplementary Note 1, for details. Directions are given in the pseudo-cubic setting of the perovskite structure.) The crystal axes are **b**=[011], which will be shown to coincide with the polar axis, and **a**=[100] and **c**=[0–11], which define the plane where the conduction charge is largely confined. Our simulation supercell comprises two five-layer blocks along **b** and is compatible with all structures of interest here. To identify the ground state, we start from the high-symmetry structure (*Immm* space group, Fig. 1a) and condense all its unstable distortions as obtained from a Hessian analysis. (See the Methods for details of the first-principles simulations.) For La-5517, the ground state is CS *Pmnn* (Fig. 1b), barring the existence of polarization. We also simulate the experimentally proposed structure of ref. 13, but find it to be a high-energy unstable configuration.

Inspired by the observation that perovskites where Bi^3+^ replaces La^3+^ tend to be ferroelectric due to Bi^3+^'s tendency to form low-coordination complexes with neighbouring oxygens^14^,^15^, we explore symmetry breaking in Bi-5517, obtained by replacing all La atoms with Bi's. As in La-5517, the *Immm* phase is a high-energy saddle point. However, at variance with La-5517, the *Pmnn* structure is also a saddle point for Bi-5517. We then condense the unstable distortions of this phase, and identify as lowest-energy solution a structure with the non-CS *Pm2*_*1*_*n* space group (Fig. 1c). We computed the Hessian for this *Pm2*_*1*_*n* structure and confirmed it to be a minimum of the energy. The symmetry breaking distortion in the *Pm2*_*1*_*n* phase can be appreciated by looking at the Bi's in the central layer (Bi_c_ in Fig. 1): although they remain at high-symmetry CS positions in both *Immm* and *Pmnn*, they move off-centre in *Pm2*_*1*_*n*, thus breaking the (011) mirror plane and yielding a symmetrywise ferroelectric structure.

**Figure 2:**
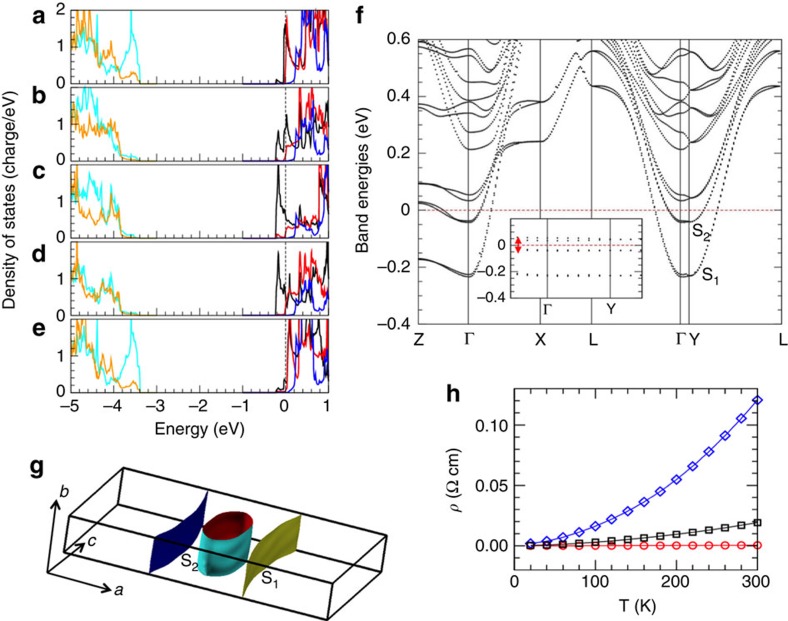
Main features of the electronic structure of Bi_5_Ti_5_O_17_. (**a**–**e**) Orbital- and atom-resolved DOS of *Pm2_1_n* Bi-5517. The five panels correspond to the five TiO (011) layers in one slab: (**c**) corresponds to the middle layer, (**a**,**e**) to the layers at the stacking, (**b**,**d**) to the intermediate layers. For each panel, the DOS is split in individual orbital contributions, indicated with different colours (only the most significant orbital components are displayed): Ti *d*_*xy*_ (black lines), Ti *d*_*xz*_ (red lines), Ti *d*_*yz*_, (blue lines), O *p* ligand parallel to the (011) planes (orange lines), and O *p* ligand orthogonal to (011) planes (cyan lines). For the *t*_2g_ orbitals, *x*, *y* and *z* correspond to the [100], [010] and [001] directions, respectively. (**f**) Band structure in the surrounding of the conduction band bottom. Inset: zoom along the Γ-Y direction. S_1_ and S_2_ label the two occupied conduction bands, and the red-arrowed line the gap (Δ*E*=0.1 eV) along Γ-Y for *k*_x_=0. (**g**) Calculated Fermi surfaces. The bounding box indicates the Brillouin zone used for the calculations, and the Cartesian axes are indicated by the arrows. S_1_ and S_2_ label the Fermi surfaces corresponding to the two occupied conduction bands. S_1_ is composed of two disconnected sheets (in green and violet), which are flat along *b* and slightly bumped along *c*. S_2_ is a single flat tube (cyan) of ellipsoidal section parallel to *b* and rounded along *a*. (**h**) Resistivity *ρ* versus temperature calculated along the supercell axes: **a**=[100] (red circlets), **b**=[011] (blue diamonds), **c**=[0–11] (black squares).

As regards the electronic structure, Bi-5517's *Pm2*_*1*_*n* phase is clearly metallic, as can be seen in Fig. 2a–e from the atom- and orbital-resolved DOS. We have two conduction electrons per primitive cell (that is, a density of 3 × 10^21^ cm^−3^), with *E*_F_ crossing the Ti 3*d*-*t*_2g_ band manifold ∼0.4 eV above the conduction band bottom (CBB). The near-CBB DOS highlights a marked two-dimensional character, with 40% of the conduction charge confined within the central Ti layer of each block, 25% in each of the two intermediate layers and only 5% in each of the edge Ti's. The *t*_2g_ CBB is split into *d*_*yz*_ (laying orthogonally to the stacking plane, rising in energy due to reduced hopping along the stacking direction) and *d*_*xy*_/*d*_*xz*_ states (the hopping along *x* being unaffected by the stacking). *d*_*xy*_ and *d*_*xz*_ are also split: only *d*_*xy*_ has significant DOS below *E*_F_ in one of the five-layer blocks, and only *d*_*xz*_ in the other, signalling orbital ordering. Figure 2f highlights the anisotropy of the conduction bands: the two occupied bands per block are doubled, there being two blocks in the supercell; yet, the splitting due to inter-block coupling is negligible, confirming good confinement of the conduction electrons within each block. The inset of Fig. 2f shows that the bands are completely flat along the Γ-Y (stacking) direction, with no band crossing *E*_F_. The system is thus gapped at Γ along this direction, although of course it may not be for a generic *k*-point away from zone centre.

Figure 2g shows the Fermi surface (FS). The lowest-energy band S_1_ consists of two disconnected parallel sheets, and the higher S_2_ band contributes an elliptic tube. Along Γ-Y (**b** direction), the FS is very flat and resistivity is high, as shown in Fig. 2h. Along Γ-X (**a** direction), the light-mass S_2_ contributes to mobility, while S_1_ is disconnected. Finally, along Γ-Z (**c** direction), both sections contribute, but yield relatively high resistivity as the corresponding masses are much heavier than along Γ-X. As a result, the predominant low-resistivity channel is largely one-dimensional along **a**; nevertheless, the resistivity temperature dependence is that of a metal in all directions. The ordering of the resistivities is quite consistent with experiments^9^ for La-5517, and the weak insulating upturn observed in La-5517 can be reproduced by inserting small defect-like activation energies in the conductivity model (see Supplementary Fig. 2 and Supplementary Note 2 for details).

One might wonder whether the metallic *Pm2*_*1*_*n* structure could experience additional symmetry-breaking distortions (for example, of the Peierls or Jahn-Teller type) that might open a gap within the conduction band and render an insulating solution. If they existed, such gap-opening distortions would appear as (soft-mode) instabilities of the *Pm2*_*1*_*n* phase, that is, they would have negative eigenvalues of the corresponding Hessian matrix associated to them. As mentioned above, we explicitly checked that no such soft mode exists, and that the *Pm2*_*1*_*n* phase is a stable energy minimum. Hence, the metallic character of Bi-5517's non-CS ground state is robust.

### Electric polarization and self-screening in Bi_5_Ti_5_O_17_

We now tackle the calculation of the ferroelectric polarization appearing in the *Pm2*_*1*_*n* phase of Bi-5517. Let us first note that the polarization—defined as the integrated current flowing along the stacking direction when we move from the CS phase (*Immm*) to the non-CS one (*Pm2*_*1*_*n*)—can be split into contributions from ionic cores, valence electrons and conduction electrons. The first two dominate the effect and are trivial to compute by standard methods^16^,^17^: we obtain *P*_ion_=55.5 μC cm^−2^ and *P*_val_=−14.6 μC cm^−2^. In contrast, calculating *P*_cond_ is not standard. Nevertheless, we can take advantage of the localized character of conduction electrons within the Bi-5517 blocks and implement two independent approaches to calculate *P*_cond_. These approaches provide consistent results.

**Figure 3:**
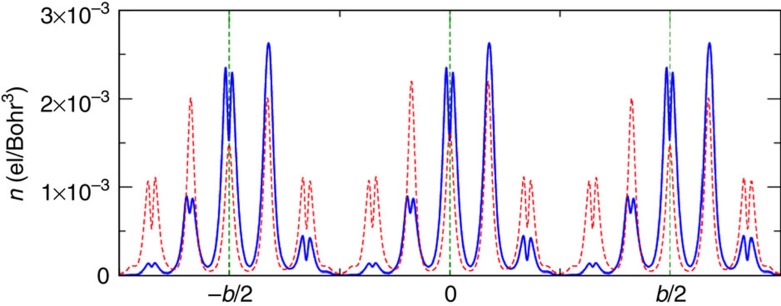
Conduction charge density of Bi_5_Ti_5_O_17_. The density is obtained by means of a planar average of the calculated three-dimensional conduction densities along the stacking direction [011]. Dashed green vertical lines indicate the position of the central Ti layer of a given block; the length *b* of the supercell along [011] includes two blocks. The blue solid line is the conduction charge density of the electrically polarized *Pm2*_*1*_*n* structure; the red dashed line is the conduction charge density calculated for a fictitious centrosymmetric system with the same cell parameters of the *Pm2*_*1*_*n* structure but internal atomic positions of the centrosymmetric *Immm* structure. The density is first calculated on the Fast–Fourier Transform grid, and then interpolated on a ultra-dense grid to reduce real-space integration errors. The polarization in the *Pm2*_*1*_*n* phase points to the right, whereas it is perfectly vanishing for the centrosymmetric phase.

First, we compute *P*_cond_ from the dipole associated to conduction electrons within a five-layer block in Bi-5517. Figure 3 shows the planar-averaged conduction charge of the *Pm2*_*1*_*n* phase, as well as that of a CS system with *Pm2*_*1*_*n* cell parameters and *Immm* atomic positions. The *Pm2*_*1*_*n* phase displays an evident inversion symmetry breaking; a dipole appears within each block and we obtain *P*_cond_=−4.0 μC cm^−2^. Note that, again, a significant two-dimensional (2D) charge confinement is apparent in Fig. 3, and this strategy to compute *P*_cond_ would be exact if the conduction charge were strictly confined within the blocks.

Alternatively, we can compute *P*_cond_ using a modified version of the Berry phase formalism. As the occupied conduction bands are rather flat along the Γ-Y direction of the Brillouin zone (the reciprocal-space signature of confinement), we can generalize the usual formulation to allow for changing numbers of contributing bands at different *k*-point strings (see the Methods for details). We eventually obtain *P*_cond_=−7.5 μC cm^−2^, which we deem in reasonable agreement with our estimate above.

Figure 3 also reveals a fascinating effect, namely, how the mobile carriers rearrange within each of the five-layer blocks to screen the field created by the local dipoles resulting from the CS-breaking displacements of the central Bi cations (see the respective enhancement and decrease in electron density on the right and left sides of the Bi_c_ planes). Remarkably, the ferroelectric instability persists in spite of this self-screening mechanism, contradicting the general notion that an abundance of mobile carriers should prevent any such polar distortion. This result highlights the difference between our material (whose ferroelectric phase has a local, chemical origin associated to the Bi–O bonding) and compounds such as BaTiO_3_ (where the ferroelectric instability relies on the action of dipole–dipole interactions^18^ that are strongly weakened by screening charges^5^). Note that the difference in behaviour between chemically driven and dipole–dipole-driven ferroelectrics is well illustrated by the predicted response of prototypical compounds such as BiFeO_3_ (similar to Bi-5517 in that Bi-O bonding dominates the ferroelectric distortion^15^,^19^,^20^) and BaTiO_3_ to electron doping and the accompanying metallization: the doping is not detrimental to the polar distortion in the former^21^, but annihilates it in the latter^5^.

Hence, our calculations indicate that the *Pm2*_*1*_*n* phase of Bi-5517 has a spontaneous polarization of ∼35 μC cm^−2^, which is in the same league as the most common ferroelectric perovskites (for example, 30 μC cm^−2^ for BaTiO_3_). Ferroelectricity largely originates from Bi^3+^ cations moving off-centre in the perovskite framework. This displacement is invertible with respect to the (011) plane, so that switching between two equivalent polar states is possible symmetrywise. The computed ferroelectric well depth of 0.31 meV Å^−3^ suggests a critical temperature upward of 500 K.

## Discussion

So far, we have shown that metallicity coexist with zero-field polarization in Bi-5517. Is it possible to switch the polarization of this ferroelectric metal? Can a finite Bi-5517 sample sustain a finite field, as would be required to switch its polarization? For Bi-5517 in the usual capacitor configuration with metallic electrodes, one may expect the bias to induce a current rather than to act on the polar distortion. For Bi-5517 cladded within insulating layers, current flow is precluded by construction (neglecting tunneling), but mobile carriers should screen an applied bias, and leave the CS-breaking distortion unaffected. It turns out that Bi-5517 is quite at odds with this reasonable expectation. We show this by studying a superlattice (SL) of alternating Bi-5517 (one primitive cell, ∼31 Å thick) and Bi_2_Zr_2_O_7_ (BZO-227, *n*=4 of the same family; layer ∼26 Å thick) layers. BZO-227 acts as a cladding insulator providing seamless stoichiometric continuity on the A-cation site as well as effective confinement of the conduction electrons within Bi-5517 (see Supplementary Fig. 3 and Supplementary Discussion for details). We compare a SL where Bi-5517 is non-CS (starting from the *Pm2*_*1*_*n* bulk phase) with a reference SL that is a suitable symmetrization of the non-CS one (which yields a *Pmnn*-like structure for the Bi-5517 layer).

**Figure 4:**
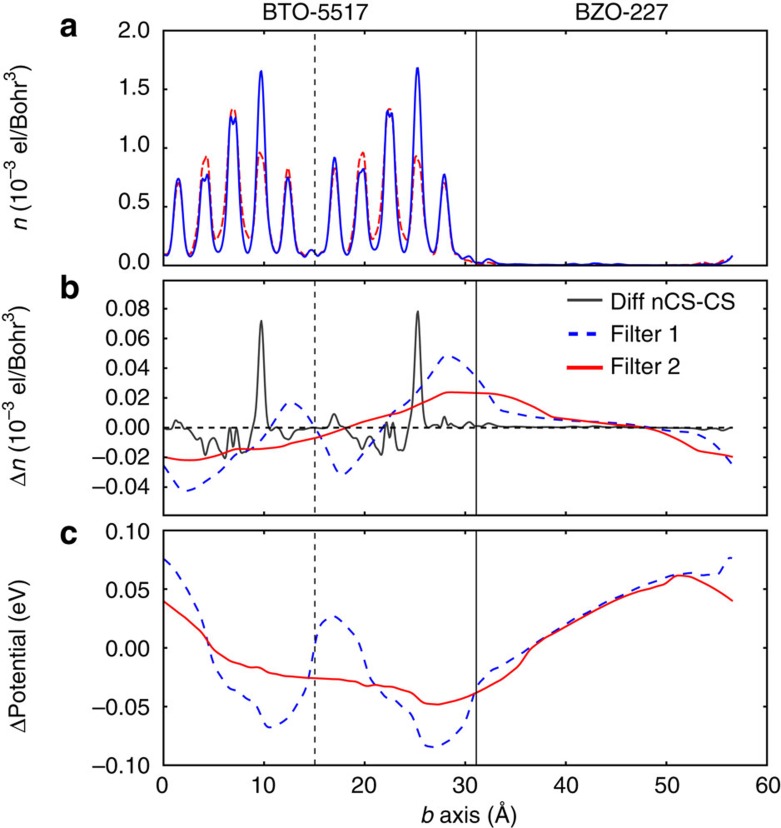
Potential and conduction charge density of Bi_5_Ti_5_O_17_/Bi_2_Zr_2_O_7_ superlattices. Bi-5517 is on the left, BZO-227 on the right, separated by vertical solid lines; dashed vertical lines mark the two Bi-5517 blocks. Horizontal line in **b** is the zero density baseline. (**a**) The planar-averaged conduction density of SL with non-CS (blue solid line) and CS (red dashed) Bi-5517 is shown. (**b**) Density differences of SL between the non-CS and CS cases in three variants are shown: planar-averaged (grey, thin, solid) reduced by a factor 10; filtered with filter [2*d*,3*d*] (labelled 2; red, thick, solid); filtered with filter [2*d,d*] (labelled 1, blue, thick, dashed). See the Methods for details. (**c**) Filtered difference of the non-CS and CS potentials (same filters and line convention as panel **b**) is shown. Note that the substantial (2 eV) interface band offset of Bi-5517 to BZO-227 cancels out in the potential difference (**c**), but leads to efficient confinement (**a**) of the conduction charge inside Bi-5517. The polarization of the non-CS phase points to the right.

Figure 4 shows planar and filter averages^7^ (see the Methods) of the potentials and conduction densities of the two SLs, and their differences. The key result is the sizable depolarizing field *E*_dep_∼20 MV m^−1^=0.02 GV m^−1^, which we estimate from the potential slope in the central region of Bi-5517 (see Fig. 4, bottom, for details). Thus, the mobile charge dominates the screening process, but is unable to screen out entirely the polarization-induced field. (This residual depolarizing field increases further if the number of mobile electrons is reduced by hole injection; see Supplementary Fig. 4 and Supplementary Discussion for details.) The difference between the non-CS and CS conduction densities (Fig. 4, top and centre panel) clearly shows, first, the local self-screening within each five-layer block already observed in the bulk case; and second, a net charge imbalance—with negative and positive carriers accumulating, respectively, at the right (Bi-5517/BZO-227) and left (BZO-227/Bi-5517) interfaces—that acts against the polarization-generated field. Although the overall self-screening response is incomplete, it is still amply sufficient to stabilize the mono-domain polar state even under such unfavourable electrical boundary conditions. (Owing to the ∇*D*=*ρ*_free_=0 condition across the interface, the mono-domain configuration of thin ferroelectric layers in a ferroelectric–dielectric SL is generally unstable; see Supplementary Discussion for details.) Indeed, explicitly relaxing the non-CS SL, we find that the Bi-5517 layer is almost identical to the polar bulk phase, which confirms the stability of the mono-domain configuration.

The switching of polarization, and more generally the response to an applied field, in Bi-5517 is a non-trivial problem. However, our finite Bi-5517 cladded layer is in an open-circuit configuration where, because of the insulating BZO-227 layers, no current flows; in this case, a plausible argument can be made for the switching. Consider the situation shown in Fig. 4, where the polarization (mostly due to ions) points towards the right side of the layer. The layer screens the polarization-induced field incompletely, so that there is a residual depolarizing field that points from right to left and acts against the polarization. The incomplete screening in itself implies that the layer is effectively a dielectric medium with finite low-frequency, low-wavevector dielectric function. Hence, it is natural to conclude that, upon application of a field exceeding the finite screening ability of the Bi-5517 layer, the polarization will switch. Pictorially, an external field pointing towards the left will push cations (oxygens) to the left (right), whereas valence electrons and mobile conduction electrons go to the right. Yet, the latter response is necessarily limited by the Bi-5517 layer's modest stock of mobile charges and the finiteness of the system, and hence the screening will be incomplete. Note that, in contrast, ferroelectric switching seems less likely in a bulk Bi-5517 sample; in that case, the reservoir of mobile carriers is essentially unlimited, and the details of the dynamical response of electrons and lattice to the external bias will probably become critical to decide whether switching can be achieved or not.

As just shown, even a unit cell of single-domain Bi-5517 is polarized and sustains a polarization-generated field. Bi-5517 thus contradicts the natural assumption that, in the nanometric-film limit^6^, polarization can never survive its own depolarization field. Indeed, Bi-5517 stands apart from any other known ferroelectric material because of the coexistence of a localized and strong polar instability (driven by the formation of Bi–O bonds) and a self-screening mechanism that does not prevent the chemically driven polar distortion but does partly cancel the corresponding depolarizing field. In this context, Bi-5517 is akin to so-called hyper-ferroelectrics^22^, whereby soft LO phonons are associated to a large high-frequency dielectric constant and small Born dynamical charges (a feature generally barred by the large gaps and Born charges characteristic of prototypical ferroelectric perovskites). In this sense, Bi-5517 behaves as a limiting case of hyper-ferroelectric, and might thus be considered an instance of self-screened hyper-ferroelectric metal.

In conclusion, we have designed a Bi-based layered-perovskite titanate that presents native metallicity—in the form of a conductive low-dimensional electron gas—and, simultaneously, complies with the requirements of a regular switchable ferroelectric. Besides its conceptual significance, and the fundamental interest of further characterizing the behaviour of Bi-5517 and related materials, our finding opens interesting perspectives for innovative applications. Intriguing possibilities range from the fields of photovoltaics (as a metal, Bi-5517 can be expected to be a good absorber that, simultaneously, features a built-in driving force to separate electrons and holes) to electronics (Bi-5517 may be expected to behave as a heavily n-doped semiconductor strongly responsive to applied fields) or spintronics (there are obvious strategies to construct a spin-polarized, multiferroic version of our ferroelectric metal). We thus hope our work will motivate further investigations of this compound and related ones based on similar strategies to achieve the co-existence of ferroelectricity and metallicity.

## Methods

### Simulation details

Our calculations are performed at the first-principles level within the local density approximation^23^,^24^ to density-functional theory and the projector-augmented wave scheme^25^ to treat the interaction between ionic cores and valence electrons, as implemented in the first-principles package VASP^26^,^27^,^28^,^29^. The following electrons are explicitly considered in the simulations: Ti's 3*s*, 3*p*, 3*d* and 4*s*; La's 5*s*, 5*p*, 5*d* and 6*s*; Bi's 5*d*, 6*s* and 6*p*; O's 2*s* and 2*p*. The electronic wave functions are represented in plane-wave basis truncated at 500 eV. For all self-consistency and force calculations, Brillouin zone integrals are computed on the k-point 6 × 1 × 5 grid, reflecting the elongated shape of the cells (*a*≈3.9 Å, *b*≈31.0–55 Å and *c*≈5.4 Å; details are given in Supplementary Table 1.)

For the analysis of the electronic structure, we also use the variational pseudo-self-interaction-corrected density-functional approach^30^,^31^, which uses ultra-soft pseudopotentials^32^ with plane-wave cutoff of 476 eV. DOS calculations use 12 × 4 × 8 grids for Brillouin zone integration.

For transport, we use two approaches: first, the Bloch–Boltzmann transport theory^33^ as implemented in the BoltzTraP code^34^, interpolating the band structure over a 30 × 14 × 22 *ab initio* calculated k-point values; second, an effective-mass band model that allows an easy inclusion of localized states below the mobility edge. In both cases, the relaxation time needed by Bloch–Boltzmann is calculated by analytic modelling, including the most important scattering contributions (that is, electron-phonon and impurity scattering). The model was previously applied to describe several low-dimensional systems involving titanates, with satisfactory results^35^,^36^,^37^,^38^.

### Polarization of metals with confined conduction charges

To compute the electronic polarization contribution from the conduction electrons, we use a modified Berry phase^16^,^17^,^39^ approach. In its standard version, this approach shows that polarization in crystals is the integrated current flowing through the system as atoms displace from the CS (*λ*=0) to the non-CS phase (*λ*=1):


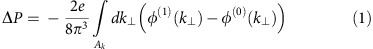


where *k*_⊥_spans the Brillouin zone section, of area *A*_*k*_, orthogonal to the polarization direction, and *ϕ* is the Berry phase of the Bloch wavefunctions:


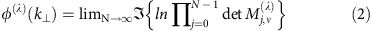


with


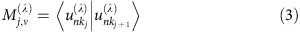


where the 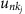
's are periodic parts of the Bloch wavefunctions, *v* the number of bands, *n*=[1, *v*] and *m*=[1, *v*] band indexes, and *k*_*j*_ runs over a string of *N* discrete points from Γ to *G*_||_, that is the shortest *G*-vector in the direction parallel to the polarization.

In general, the above expressions cannot be applied to a metal, as the Berry phase is well defined (that is, gauge-dependent by unitary rotation) as long as the bands that contribute to the matrix in the above equation are an isolated subgroup (typically the whole valence band manifold of an insulator). Clearly, for a metal, this condition does not hold, as *v* depends on *k*, that is, the number of occupied bands change with *k*. However, if the system has flat bands along a specific direction (this applies to Bi-5517 since electrons are quite localized within each 5-unit block along the ***b*** axis), then the number of occupied bands only changes with *k*_⊥_, but not along the string, that is, no band crosses the Fermi level along the k-space string parallel to the insulating direction. It follows that 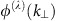
is well defined for any *k*_⊥_, while *v* can change with *k*_⊥_ with the constraint


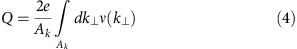


on the total electron charge *Q*. In practice 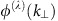 may fluctuate widely with *k*_⊥_ due to the change in the number of bands contributing to the string, and in turn the 2D average in the above equation may be slowly convergent. The considerable computing effort required to calculate the Berry phase in large-size systems (such as Bi-5517) suggests adopting a strategy to minimize the contribution of the variable-band-number part. In the specific case of Bi-5517, the latter contribution is limited to a few conduction bands, well separated by a large band gap from the valence bands. We therefore first calculate separately the polarization due to valence bands, which typically converges rapidly over a limited set of *k*_⊥_ points. For the conduction bands, the polarization is then calculated as the 2D average of the renormalized phase


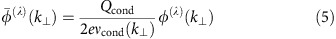


where *Q*_cond_ is the conduction charge and *v*_cond_ the number of occupied conduction bands at *k*_⊥_. With this choice, each *k*-string contribution is renormalized to the same number of electrons, and fluctuates much less with *k*_⊥_, reducing the effort needed to converge the calculation. Further, in this approximation, even partial band occupancies—as it would correspond to *k*_⊥_ points at the FS—can be handled by allowing fractional 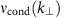 values.

### Filters

To analyse the potential and charge density in the SLs, we apply well-known averaging processes, described, for example, in refs 7, 40. The filter average features prominently in Fig. 4 of the paper, and is defined as


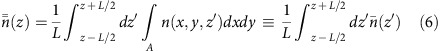


with *n* a function (for example, the charge density). It is a one-dimensional square-wave filter of the planar average (implicitly defined by the second equality, and calculated over a sectional area *A*) over a window of fixed width *L*, which is its defining parameter. If *L* is the microscopic periodicity length (assuming such periodicity exists), all microscopic oscillations in the planar average are eliminated. This basically amounts to filtering away all the Fourier components corresponding to microscopic oscillations.

However, the typical potential or charge density in our system has a rather wide spectrum in wavevector space, and filtering all components would entail a complete loss of information. So, in practice, we choose filters such that microscopic oscillations are reduced significantly after a couple of passes at most. We find that applying the filter twice, with *L*=*d* and 2*d*, or with *L*=3*d* and 2*d*, gives the best results in terms of oscillation removal, *d* being the average interplanar distance.

## Additional information

**How to cite this article:** Filippetti, A. *et al*. Prediction of a native ferroelectric metal. *Nat. Commun.* 7:11211 doi: 10.1038/ncomms11211 (2016).

## Supplementary Material

Supplementary Information Supplementary Figures 1-4, Supplementary Table 1, Supplementary Notes 1-2, Supplementary Discussion and Supplementary References
