# Supplementary material for: Prediction of a native ferroelectric metal
Source: Nat Commun. 2016 Apr 4;7:11211. doi: 10.1038/ncomms11211 (PMC4822038; doi:10.1038/ncomms11211)
Supplement: Supplementary Information — Supplementary Figures 1-4, Supplementary Table 1, Supplementary Notes 1-2, Supplementary Discussion and Supplementary References [file ncomms11211-s1.pdf]

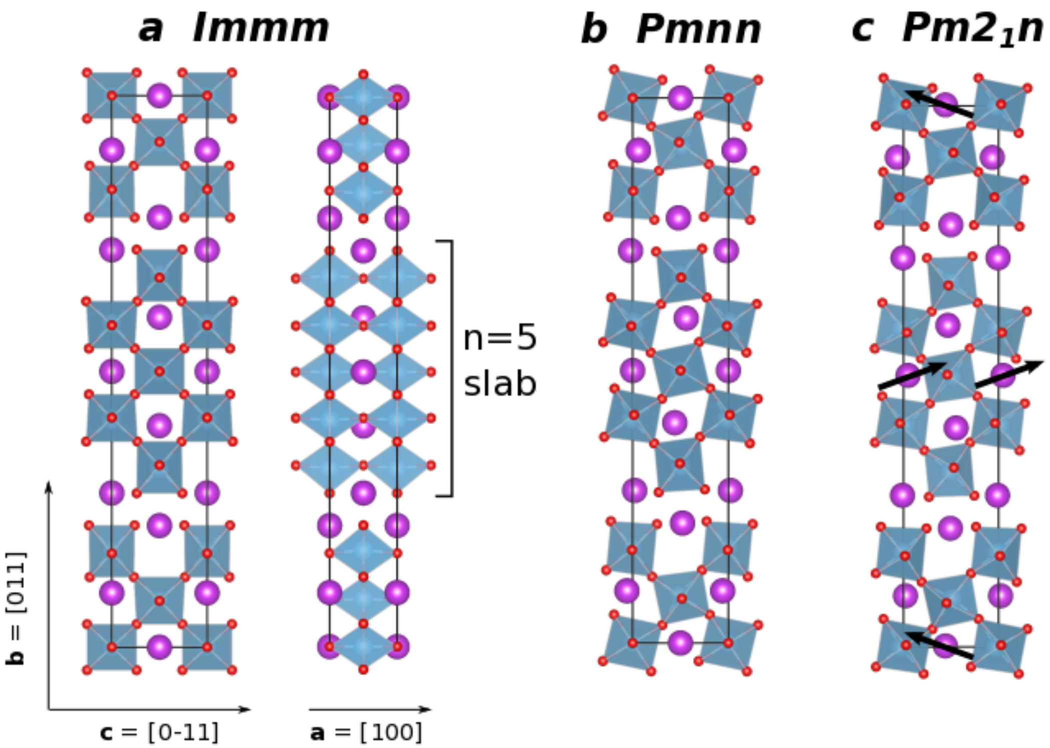

Supplementary Figure 1 | Structural detail of the $Bi_5Ti_5O_{17}$ simulations. We show the *Immm* (**a**), *Pmnn* (**b**), and $Pm2_1n$ (**c**) structures of layered *n*=5 perovskite, as obtained from first-principles relaxations. The directions of the supercell vectors are indicated in (**a**); the Cartesian setting is chosen to coincide with the pseudo-cubic axes of the perovskite lattice. In (**c**) we indicate the off-centering displacement of the Bi cations in the central layers. Large violet balls stand for Bi atoms, small red balls for oxygens, green polyhedra for the TiO_6 groups. Most relevant atomic distortions indicated with arrows.

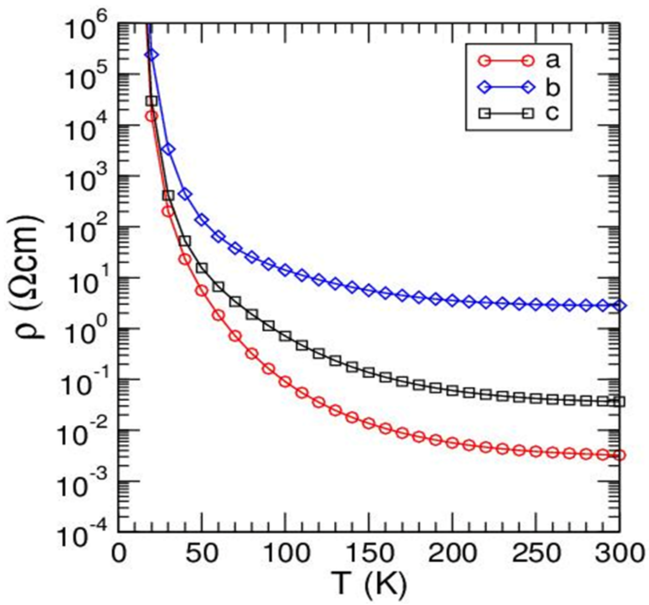

Supplementary Figure 2 | Resistivity in presence of defects. Electric resistivity vs. temperature calculated for Bi-5517 by effective mass modeling (described in the text). The resistivity components along the Cartesian directions are shown, wehre **a** lies along [100], **b** along [011], and **c** along [0-11].

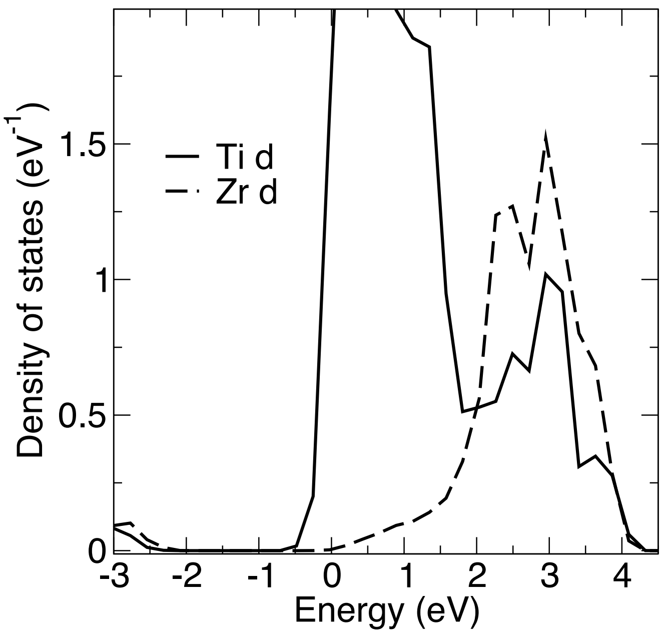

Supplementary Figure 3 | Local density of states of the $Bi_5Ti_5O_{17}/Bi_2Zr_2O_5$ superlattice. We show the projections on the Ti and Zr atoms adjacent to the interface on the right side of Fig. 4 of the main text (the low potential side).

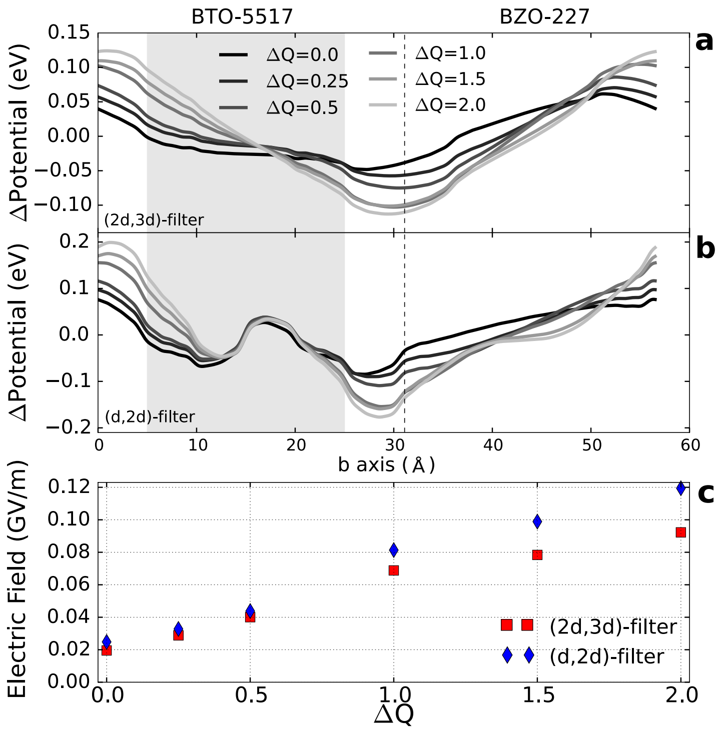

Supplementary Figure 4 | Superlattice results as a function of hole doping. (**a**) and (**b**): filter averages of the potential difference between the non-CS and CS states of the Bi-5517/BZO-227 superlattice for removed charges from 0 to 2 (the shade of gray is lighter and the thickness increases along the series). Clearly the slope increases with the removed charge. (**c**): field value as a function of the removed charge for the two different filters. The gray-shaded area indicate the filtering region used to calculate the potential average. Filters (d,2d) and (2d,3d) are defined in the Methods section.

	La-5517				Bi-5517			
Phase	E	a	b	c	E	a	b	c
$Immm$	0.74	3.885	31.276	5.439	1.55	3.863	31.461	5.435
$Pmnn$	0	3.912	30.805	5.422	0.21	3.902	30.975	5.418
$Pm2_1n$	–	–	–	–	0	3.890	31.100	5.461
Expt		3.929	31.466	5.532	–	–	–	–

Supplementary Table 1 | Details of investigated phases. Energy (eV/cell) and lattice constants (Å) of various phases of La-5517 and Bi-5517. *a*, *b*, and *c* are the lengths of the crystal vectors, which are parallel to the [100], [011], and [0$\bar{1}$1] directions, respectively. The ground states are *Pm2₁n* for Bi-5517 and *Pmnn* for La-5517. Other energies are referred to these ground states. Experimental lattice parameters[1] for La-5517 are also reported for comparison.

Supplementary Note 1

For our simulations of bulk $La_5Ti_5O_{17}$ and bulk $Bi_5Ti_5O_{17}$, we employ the 54-atom cell depicted in Supplementary Figure 1a, which contains 2 slabs with n=5 [011]-oriented perovskite-like planes *per* slab. This cell is the conventional one for the high-symmetry reference structure (*Immm* space group) depicted in Supplementary Figure 1a, and the primitive cell for the lower-symmetry structrues (*Pmnn* in Supplementary Figure 1b and $Pm2_1n$ in Supplementary Figure 1c) discussed in this work. Note that, in the *Immm* phase, the two slabs are related by a (**a**+**b**+**c**)/2 translation, where **a**, **b**, and **c** are the lattice vectors indicated in Supplementary Figure 1a. The distortion connecting the *Immm* and *Pmnn* structures essentially consist of rotations of the O_6 octahedra about the **a** axis, as can be appreciated by comparing Supplementary Figures 1a and 1b, and is indicated in Fig. 1 of our article. The phase of the rotations is shifted from one slab to the other; as a consequence, the primitive cell doubles. Then, the distortion connecting the *Pmnn* and $Pm2_1n$ structures has a polar character, **b** being the polar axis. The corresponding polarization is largely dominated by the off-centering of the Bi cations at the central layer of the slabs, as discussed in our paper. Additionally, by comparing Supplementary Figures 1b and 1c, in-plane displacements of the cations, along the **c** direction, can be easily appreciated. Symmetrywise, such displacements constitute a **c**-polarized distortion within each slab; however, as can be seen in Supplementary Figure 1c, these distortions change sign when we move from one slab to the next, the net effect being null. (Thus, along **c** we have an anti-ferroelectric pattern of sorts.) For completeness in Supplementary Table 1 we report some structural and energetic details of the various structures in the two materials.

Supplementary Note 2

Figure 2d of the main text shows that the resistivity is anisotropic and metal-like. Resistivity experiments[1] for La-5517 (nearly isostructural to our newly designed Bi-5517, and the closest existing material in many respects[2]) suggest that the resistivity components are insulator-like over significant temperature intervals. The resistivity is activated, with several different small activation energies at play, indicating that the upturn is probably due to defects of some kind. This situation can be described theoretically considering an effective-mass two-band model including a light-mass (0.7 m_e) t_{2g} band edge and a single localized state lying 40 meV lower, contributing to current by thermal activated hopping. The calculated resistivities along the crystal axes, shown in Supplementary Figure 2, are anisotropic and have an insulating upturn quite similarly to experimental ones. This corroborates the attribution of the insulator-like resistivity upturn in such low-density, flat Fermi-surface metals to low-activation-energy defects.

Supplementary Discussion

In the following we further discuss depolarizing fields in our investigated materials, commenting on similarities and differences with regular ferroelectrics.

Depolarizing field: geometry and confinement. The main issue in the assessment of the depolarizing field in Bi-5517 is the termination of the finite system. We checked that a slab of a normal insulating ferroelectric ($PbTiO_3$ in the (001) direction) in vacuum has the same depolarizing field with symmetric or asymmetric surface terminations. This need not apply to Bi-5517, given the presence of free charge. The symmetric BiO surface-terminated slab, obtained adding vacuum above and below the primitive cell, has essentialy zero residual field, i.e no macroscopic dipole; this is due to the conduction charge being almost entirely located at surface states, and therefore screening the polarization charge very effectively. The asymmetric termination with a TiO_2 layer on one side and a BiO on the other leads to a large field: however, this results again from conduction charge bound into surface states on the Ti-terminated surface, rather than from screened polarization. This is also confirmed by calculations in another asymmetric surface termination, whereby we find a large field opposite to that expected from polarization.

This motivates us to extract the properties of a finite Bi-5517 system using a Bi-5517/insulator superlattice. We choose $Bi_2Zr_2O_7$ (BZO-227) as our cladding insulator. This material is non-polar in the configuration we impose on it, although it turns out to be ferroelectric with polarization along the **c** axis when relaxed as stand-alone bulk. BZO is also BTO-stoichiometric on the A site, thus virtually eliminating the possibility of interface-state related fields.

BZO-227 provides a good confinement of the conduction electrons within Bi-5517, which is desirable in the present case. Examining the locally-projected density of states (DOS), we find that at the BTO/BZO interface there is essentially zero valence band offset, but a sizable conduction band offset. As shown in Supplementary Figure 3, the centroid of the d DOS of the Zr adjacent to the interface is about 2 eV higher than the d DOS of the Ti just opposite to it across the interface. (We consider the d DOS since the conduction band is mostly of d character of the octahedrally coordinated cation.) There is a tail of Zr DOS overlapping Ti's DOS, due to a single orbital presumably involved in bonding across the interface. Thus, it seems fair to extract the conduction offset by a linear extrapolation of the two DOS to zero following the slope on the low energy side of the main peaks. This gives an offset of about 2.0 to 2.2 eV. In Fig. 4c of the main paper this offset is not directly visible as it cancels out in the potential difference, but is indirectly visible in the confinement of the conduction charge inside Bi-5517 shown in Fig. 4a.

Depolarizing field vs density of mobile charge. Any reduction of the conduction charge (2 electrons per cell in Bi-5517) should cause an increase of the depolarizing field. This reduction may be produced by doping or field-effect injection; for example, Ca substitution of Bi at the 10% level (i.e. two Bi-5517 units become $CaBi_9Ti_{10}O_{34}$) will reduce the conduction charge in the unit cell to 1 electron. Since doping is difficult to simulate and may lead to unintended consequences (such as modifying the polarization, etc.), we study the effect of conduction charge removal for the same superlattice as before, simply subtracting by hand a certain amount of

charge ΔQ. (The ions are kept clamped for simplicity; as mentioned in the paper, the polar distortion is unaffected by relaxation.) The charge is effectively removed from within Bi-5517, and neutrality is maintained by a uniform compensating background that spreads over the whole cell. The spurious potential thus induced is eliminated automatically by taking the difference of the potentials of non-CS and CS superlattices. We can thus compare the filtered averages of the potential for various values of ΔQ all the way up to 2 electrons, i.e. to zero conduction charge remaining, and extract the field in the superlattice as a function of removed charge.

In Supplementary Figures 4a-b we report the potential difference between non-CS and CS superlattices for various values of ΔQ, for both the filters mentioned in the previous Section, in the two top panels. The [d,2d] filter, in particular, highlights the local screening within each block of Bi-5517. In Supplementary Figure 4c we show the fields extracted from the total potential drop across the gray-shaded regions for both filters. Clearly, the values are quite similar in both cases. In the limiting case of 2 electrons removed, the field is limited by the gap to about 0.5 GV/m. The valence electron screening brings it down further to about 0.1 GV/m, for an effective valence dielectric constant of 5.

Ferroelectricity in a finite field. It is known[3] that the energy associated with the depolarizing field may destroy ferroelectricity in a finite system. We checked that is the case for a $PbTiO_3$ slab containing four Ti units. The unscreened bulk polarization charge (0.8 C/m^2) would generate a field of 90 GV/m and a corresponding energy of 100 eV in the simulation cell. With ions clamped in the ferroelectric configuration, the purely electronic response reduces the field to 5 GV/m, i.e. a 0.3 eV field energy. Since the ferroelectric well depth of $PbTiO_3$ is about 0.08 eV per Ti, this field energy is sufficient to remove ferroelectricity. Indeed relaxing the ions we recover the paraelectric geometry.

This is not an issue for Bi-5517. The bare $\mathbf{\Delta}P$ field is 37 GV/m (field energy 51 eV in the SL cell), but the screened field is 0.02 GV/m, and hence the field energy becomes negligible and the system is comfortably on the ferroelectric-stable side.

Supplementary References

1. **Lichtenberg, F., Herrnberger, A., Wiedenmann, K. & Mannhart, J.** Synthesis of perovskite-related layered $A_nB_nO_{3n+2} = ABO_x$ type niobates and titanates and study of their structural, electric and magnetic properties. Prog. Sol. St. Chem. **29**, 1 (2001)

2. **Nanamatsu, S., Kimura, M., Doi, K., Matsushita, S. & Yamada, N.** A new ferroelectric: $La_2Ti_2O_7$. Ferroelectrics **8**, 511 (1974).

3. **Meyer, B. & and Vanderbilt, D.** Ab initio study of $BaTiO_3$ and $PbTiO_3$ surfaces in external electric fields. Phys. Rev. B **63**, 205426 (2001).
